# Different Colours of Polycythemia Vera: A Case Series

**DOI:** 10.7759/cureus.90913

**Published:** 2025-08-24

**Authors:** Naveenkumar Viswanathan, Sahasyaa Adalarasan, Sandhiya T, Yogesh S

**Affiliations:** 1 General Surgery, Cwm Taf Morgannwg University Health Board, Merthyr Tydfil, GBR; 2 College of Medicine, Madras Medical College, Chennai, IND; 3 Internal Medicine, Madras Medical College and Rajiv Gandhi Government General Hospital, Chennai, IND

**Keywords:** different presentation, jak2 mutation, myeloproliferative, polycythemia, polycythemia vera (pv)

## Abstract

Polycythemia vera (PV) is a myeloproliferative neoplasm characterized by increased red blood cell mass, often with leukocytosis and thrombocytosis, predisposing to thrombotic and hemorrhagic events. We report three cases with varied presentations: a 38-year-old man with headaches, dizziness, blurred vision, and fatigue; a 54-year-old woman with acute myocardial infarction; and a 60-year-old man with acute ischemic stroke. All had elevated hemoglobin/hematocrit, low erythropoietin, and positive JAK2 mutation. Management with phlebotomy, hydroxyurea, and supportive measures led to good recovery. These cases illustrate the spectrum of PV manifestations, emphasizing early diagnosis and treatment to reduce morbidity and mortality.

## Introduction

Polycythemia vera (PV) is a chronic myeloproliferative neoplasm (MPN) characterized by clonal proliferation of hematopoietic stem cells, leading to an abnormal increase in red blood cell (RBC) mass. The term polycythemia refers to an increase in the total amount or volume of RBCs in the body. When there is a rise in the concentration of erythrocytes, it is termed erythrocytosis, which can be classified as absolute, due to an increase in red cell volume or mass, or relative/spurious, caused by a reduction in plasma volume. Absolute polycythemia is further divided into primary and secondary types. PV is the classic example of primary absolute polycythemia. Secondary absolute polycythemia can result from conditions such as chronic lung disease-induced hypoxia, carboxyhemoglobinemia related to smoking, and renal cell carcinoma.

In PV, symptoms frequently arise from blood hyperviscosity caused by a marked increase in blood cell components, which leads to sluggish blood flow, reduced oxygen delivery, and a predisposition to both thrombotic and hemorrhagic complications [1]. The disease may have an insidious onset, and early symptoms such as headache, dizziness, vertigo, tinnitus, visual disturbances, intermittent claudication, and transient ischemic attacks are often nonspecific. Splenomegaly and facial plethora may also be noted on examination, reflecting increased red cell mass and extramedullary hematopoiesis.

Complications can be broadly categorized into bleeding and thrombotic events. Bleeding complications, occurring in about 1% of cases, may involve nosebleeds, gum bleeding, bruising, and gastrointestinal bleeding. Thrombotic complications, also seen in approximately 1% of cases, include venous thromboses such as Budd-Chiari syndrome (hepatic vein thrombosis), mesenteric vein thrombosis, and thromboembolism, with an elevated risk of stroke, myocardial infarction (MI), and other arterial thromboses [2]. Both the thrombotic and bleeding tendencies arise from complex alterations in platelet function, vascular endothelium, and blood flow dynamics.

## Case presentation

Case 1

A 38-year-old man presented with headaches, dizziness, blurred vision, and fatigue. Clinical suspicion of PV was raised due to hyperviscosity symptoms. Laboratory investigations revealed hemoglobin of 22 g/dL, hematocrit of 66%, and RBC count of 6 million/cu.mm, WBC count of 9,100/cu.mm, and platelet count of 7.4 lakh/cu.mm (Table 1). Peripheral smear showed erythrocytosis with microcytic RBC, along with leucocytosis and thrombocytosis. Serum erythropoietin (EPO) was low, and the JAK2 mutation was positive, confirming PV. He was treated with phlebotomy and hydroxyurea, resulting in significant improvement within two weeks, following which he was discharged.

**Table 1:** Results of investigations in the three patients HCT: hematocrit; TC: total WBC count; RBS: random blood sugar; ECHO: echocardiogram; LAD: left anterior descending

Parameters	Reference range	Case 1	Case 2	Case 3
Hemoglobin (gm/dL)	13.5-17.5 (M), 12-15.5 (F)	22	18.4	19.2
HCT (%)	41-53 (M), 36-46 (F)	66	55.6	58
TC (cells/mm^3^)	4000-11000	12,400	14,000	11,900
Platelets (lakh cells/mm^3^)	1.5-4.5	7.4	6.3	5.3
RBS (mg/dL)	100-140	79	110	112
Urea (mg/dL)	15-40	19	25	33
Creatinine (mg/dL)	-	0.4	0.7	1.0
T. bilirubin (mg/dL)	-	0.5	0.2	0.9
D. bilirubin (mg/dL)	-	0.2	0.3	0.4
Sodium (mEq/L)	135-145	136	142	137
Potassium (mEq/L)	3.5-5	3.5	4	3.9
Peripheral smear	-	Erythrocytosis with microcytic RBCs, leucocytosis, and thrombocytosis	Erythrocytosis with microcytic RBCs, leucocytosis, and thrombocytosis	Erythrocytosis with microcytic RBCs, leucocytosis, and thrombocytosis
Protein C	-	Normal	Normal	Normal
Protein S	-	Normal	Normal	Normal
Homocysteine (µmol/L)	5-15	8	7	9
Erythropoietin	-	Low	Low	Low
JAK2 mutation	-	Positive	Positive	Positive
ECG	-	Normal	Showing ST elevation in LAD territory	Normal
CT brain	-	Normal	Not done	Left territory infarct seen
ECHO	-	Normal	Regional wall motion abnormality seen	Normal
Coronary angiography	-	Not done	LAD stenosis	Not done
Outcome	-	Improved	Improved	Improved

Case 2

A 54-year-old woman presented with chest pain and breathlessness. ECG confirmed an acute MI. Laboratory investigations showed hemoglobin of 18.4 g/dL, hematocrit of 55.6%, RBC count of 5.8 million/cu.mm, WBC count of 14,200/cu.mm, and platelets at 6.3 lakh/cu.mm (Table 1). Peripheral smear revealed elevation in all three cell lines: erythrocytosis, leucocytosis, and thrombocytosis. Low EPO levels and a positive JAK2 mutation confirmed PV. She was managed with phlebotomy, antiplatelet agents, statins, and supportive care, with good recovery and discharge.

Case 3

A 60-year-old man with a history of alcohol use presented with symptoms of an acute cerebrovascular accident. MRI imaging confirmed a left middle cerebral artery infarct. Blood tests revealed hemoglobin of 19.2 g/dL, hematocrit of 58%, RBC count of 6.1 million/cu.mm, WBC count of 11,700/cu.mm, and platelet count of 5.6 lakh/cu.mm (Table 1). Peripheral smear showed erythrocytosis without leucocytosis or thrombocytosis. Serum EPO was low, and the JAK2 mutation was positive. He was treated with phlebotomy, hydroxyurea, and antiplatelet drugs, resulting in notable improvement and subsequent discharge. The investigations are compared and contrasted in Table 1.

## Discussion

PV is a chronic MPN characterized by the overproduction of RBCs, and frequently of WBCs and platelets. This abnormal proliferation is primarily driven by acquired mutations, most commonly in the JAK2 gene, which leads to constitutive activation of signaling pathways that bypass normal regulatory controls [3].

The JAK2 V617F mutation, found in over 95% of PV cases, activates the JAK-STAT pathway and drives the unchecked production of hematopoietic cells. A smaller subset of patients carries exon 12 mutations in JAK2. Additional genetic abnormalities, such as trisomy 8, 20q deletion, and loss of heterozygosity on chromosome 9p, contribute to disease heterogeneity and may influence progression and thrombotic risk. Ongoing research into these mutations is aiding the development of more personalized treatment strategies [4].

Clinically, PV often presents insidiously. Hyperviscosity from erythrocytosis manifests as headaches, dizziness, tinnitus, and visual disturbances, and it significantly increases the risk of transient ischemic attacks, stroke, and MI. The prothrombotic state also predisposes patients to venous thromboses, including rare presentations like Budd-Chiari syndrome. Though less frequent, bleeding complications such as epistaxis and gastrointestinal hemorrhage may occur due to platelet dysfunction. Splenomegaly, reflecting the sequestration of excess blood cells, is a common physical finding. Another hallmark symptom is aquagenic pruritus, a distressing itch triggered by contact with water, often resistant to conventional treatment and unique to PV [5].

The three cases presented in this series underscore the broad clinical spectrum of PV, from mild hyperviscosity symptoms to life-threatening thrombotic events such as MI and stroke. Each case highlights a distinct complication and emphasizes the importance of early recognition, accurate diagnosis, and risk-adapted therapy. Consistently, the presence of JAK2 mutation and low EPO levels supported the diagnosis, reinforcing the utility of these markers in distinguishing PV from secondary erythrocytosis. Ultimately, these cases reflect the complexity of PV, the need for individualized management, and the importance of vigilant follow-up even after initiation of treatment. Despite advances in therapy, patients remain at elevated risk for vascular events, underscoring the necessity for a comprehensive and sustained approach to care.

Diagnosis hinges on elevated hemoglobin, hematocrit, and red cell mass, with additional findings like leukocytosis and thrombocytosis. A low serum EPO level helps differentiate PV from secondary causes of erythrocytosis (e.g., chronic hypoxia, high altitude, EPO-producing tumors). JAK2 mutation testing is essential for confirmation. Bone marrow biopsy, though not always required, typically shows hypercellularity with trilineage proliferation [6].

First-line therapy includes phlebotomy, targeting hematocrit levels below 45% in men and 42% in women to reduce thrombotic risk [7]. For high-risk patients, typically older individuals or those with prior thrombotic events, cytoreductive therapy is added, most commonly with hydroxyurea. Newer agents like pegylated interferon-alpha are emerging for their potential to lower the JAK2 allele burden and modify disease trajectory [8]. In patients resistant or intolerant to hydroxyurea, ruxolitinib, a JAK inhibitor, is an approved alternative that offers symptomatic relief and reduction in spleen size [9]. Low-dose aspirin is routinely prescribed for thromboprophylaxis, while non-vitamin K antagonist oral anticoagulants (NOACs) may be considered in patients requiring stronger anticoagulation. While PV has a relatively favorable prognosis with appropriate management, it carries risks of progression to post-PV myelofibrosis and acute myeloid leukemia (AML) [10]. Long-term monitoring is essential. Ongoing research into targeted JAK2 inhibitors, gene editing, and advanced biologics holds promise for improved disease control and reduced toxicity [9].

## Conclusions

PV presents with a wide range of clinical symptoms and outcomes, from mild constitutional symptoms to life-threatening events such as stroke and MI. The cases reveal PV's complex pathophysiology and the significance of early and precise diagnosis through JAK2 mutation testing, hematologic profiling, and measurement of EPO levels. Effective management, particularly with phlebotomy, hydroxyurea, and supportive therapy, can improve outcomes, but the disease's risk of severe complications requires vigilant, individualized care.

These cases highlight the need for timely recognition, prompt treatment, and ongoing risk assessment to mitigate PV's thrombotic risks and improve patient prognosis. This study contributes valuable insights for improving PV management and patient quality of life through timely and targeted interventions.
